# HLA-C locus alleles may modulate the clinical expression of psoriatic arthritis

**DOI:** 10.1186/ar2097

**Published:** 2006-12-13

**Authors:** Ruben Queiro, Segundo Gonzalez, Carlos López-Larrea, Mercedes Alperi, Cristina Sarasqueta, Jose Luis Riestra, Javier Ballina

**Affiliations:** 1 Rheumatology Service, Hospital Universitario Central de Asturias (HUCA), C/Celestino Villamil s/n. 33006, Oviedo, Spain; 2 Department of Functional Biology, University of Oviedo, C/Julian Claveria s/n. 33006, Oviedo, Spain; 3 Immunology Department, Hospital Universitario Central de Asturias (HUCA), C/Celestino Villamil s/n. 33006, Oviedo, Spain; 4 Clinical Epidemiology Unit, Complejo Hospitalario Donostia, P° del Dr Beguiristain 111, 20014, San Sebastian, Basque Country, Spain

## Abstract

The aim of the present study was to evaluate the relative contribution of human leukocyte antigen (HLA)-C locus alleles in determining the risk and the clinical expression of psoriatic arthritis (PsA). One hundred PsA patients were randomly selected and grouped into three disease subsets: oligoarthritis (*n *= 40), polyarthritis (*n *= 25) and spondylitis (*n *= 35). The HLA-C locus profile of this cohort was studied by methods based on molecular biology and was compared with that of 45 patients with psoriasis vulgaris and 177 healthy blood donors from the same ethnic origin. HLA-Cw*0602 was found associated with both psoriasis (odds ratio (OR) 6.2; 95% confidence interval (CI) 3.1 to 12.5; *p *< 0.0001) and PsA (OR 6.2; 95% CI 3.6 to 10.8; *p *< 0.0001); however, this allele was equally found among the PsA subsets. HLA-Cw6-positive patients showed a longer psoriasis-arthritis latency period (*p *= 0.012). HLA-Cw*0701 was found under-represented in PsA in comparison with controls (OR 0.5; 95% CI 0.3 to 0.9; *p *= 0.04), as was HLA-Cw*0802 (OR 0.3; 95% CI 0.08 to 1; *p *= 0.05). A positive association was found between psoriatic spondylitis and HLA-Cw*0702 (OR 5.0; 95% CI 1.4 to 25; *p *= 0.01). HLA-Cw*0602 seems to confer a general risk for psoriasis, but the presence of other HLA-C locus alleles may explain an additional arthritogenic risk. HLA-C alleles may modulate some aspects of the clinical expression of PsA, but these findings need confirmation.

## Introduction

It is thought that the development of psoriatic arthritis (PsA) is related to an interaction between several genetic, immunological and environmental elements, and there is now convincing evidence to support this view [1].

Although the inheritance of psoriasis seems to be polygenic, previous studies have localized the PSORS1 (psoriasis susceptibility 1 gene) locus to the proximal MHC (major histocompatibility complex) class I region. Approximately a dozen genes in this region have so far been genetically inseparable, but very recent studies suggest that among them, human leukocyte antigen (HLA)-Cw6 itself is the true psoriasis gene [2,3]. In addition, this gene is also relevant to modulation of the clinical expression of psoriasis, as demonstrated by the fact that HLA-Cw6-positive patients show an earlier disease onset and more family aggregation than those without it [4].

Genetic factors are also important in both susceptibility to and the expression of PsA; however, genetic association studies of PsA are limited, among other factors, by its changing articular pattern over time and the known phenomenon of linkage disequilibrium between genes. The latter may explain why some HLA genes originally associated with PsA susceptibility are now being considered part of the ancestral haplotypes related to psoriasis risk rather than true associations [5]. Moreover, it has been difficult to discover whether HLA-Cw6 itself is associated with PsA, because this is the primary marker for psoriasis and most patients with PsA have cutaneous psoriasis; in contrast, some recent investigations have shown that both conditions share a 100-kilobase susceptibility region telomeric to HLA-C [6].

Few studies have investigated the potential role of HLA-C locus alleles in the clinical expression of PsA, so this study was undertaken to determine the relative contribution of HLA-C alleles to psoriasis and PsA susceptibility, and as a subsidiary purpose to evaluate whether these alleles confer some additional clinical feature on this disease.

## Materials and methods

For the purposes of the present study, 100 PsA patients and 45 patients with chronic stable cutaneous psoriasis were consecutively recruited at random from the general database of the rheumatology and dermatology departments of a tertiary care hospital. In our health care context, psoriatic patients with suspected arthritis are sent to our early arthritis clinic, where they are further evaluated to confirm or rule out the diagnosis of PsA. The PsA group was studied with a specific protocol designed for this study, which included epidemiological and demographic data, medical history, clinical features, physical examination, laboratory data (including tests for rheumatoid factor and antinuclear antibodies) and radiographs. Peripheral and axial joints were evaluated with standard methods. All synovial fluid samples were cultured and analysed for crystals. Pathogens that habitually cause arthritis were properly excluded. The PsA group was initially defined and classified as described in Moll and Wright's proposal [7] but, because of the changing nature of PsA with time, patients were classified in accordance with their predominant articular pattern seen in the previous 5 years of disease evolution. Thus, patients with four or fewer inflamed joints were labelled as having oligoarthritis; those with five or more swollen joints were defined as having polyarthritis; and patients with inflammatory back pain and bilateral grade II or unilateral grade III or more X-ray sacroiliitis were grouped as having spondylitis, irrespective of the presence of peripheral synovitis. Distal interphalangeal joint disease as well as mutilans forms were not computed as independent models but rather as a characteristic of PsA.

We performed HLA-C typing in 145 patients (psoriasis and arthritis) and in a control population of 177 random donors from our general population. DNA was isolated from lymphocytes by standard procedures. HLA-C alleles were specifically amplified with a combination of the sense primer SV1 (exon 2, codon 45) and the antisense primer SV2 (exon 3, codon 182). The spanning sequences (680 base pairs) containing the hypervariable regions of HLA-C exons 2 and 3 were used to examine the HLA-C alleles. Polymerase chain reaction conditions for the amplifications of exons 2 and 3 were 95°C for 30 seconds and 67°C for 50 seconds (50 cycles), with a initial denaturation step of 98°C for 1 minute and a final extension step of 70°C for 5 minutes. The specificity of the PCR-SSOP (polymerase chain reaction-sequence-specific oligonucleotide probes) method was checked by using B lymphoblastoid cell lines as positive controls for the HLA-C alleles.

The distribution of HLA-B27 antigen was also analysed in the three groups of PsA.

The strength of the association between HLA-C/B27 antigens and disease was calculated by odds ratio (OR), and the statistical significance of these associations was assessed with a two-tailed Fisher's exact test. To compare HLA-Cw6-positive with HLA-Cw6-negative individuals, χ^2 ^and Student's *t *tests were used to examine statistical differences, depending on the type of variables analysed.

All patients were informed about the objectives of the study, and informed consent sheets were obtained.

## Results and discussion

The main characteristics of this series are shown in Table 1. HLA-Cw*0602 was found to be associated with psoriasis (OR 6.2; 95% confidence interval (CI) 3.1 to 12.5; *p *< 0.0001) and PsA (OR 6.2; 95% CI 3.6 to 10.8; *p *< 0.0001), but it was equally distributed among the three articular categories defined for the study. HLA-Cw*0602-positive individuals showed an earlier age of psoriasis onset (23 ± 13 years) than HLA-Cw*0602-negative patients (32 ± 14 years; *p *= 0.012). The psoriasis-arthritis latency period was shorter in Cw6-negative patients (5 ± 5 years) than in Cw6-positive subjects (9 ± 7 years; *p *= 0.03). There were no other differences between patients with and without this allele. HLA-Cw*0701 was found under-represented in PsA (OR 0.5; 95% CI 0.3 to 0.9; *p *= 0.04), as well as in HLA-Cw*0802 (OR 0.3; 95% CI 0.08 to 1.00; *p *= 0.05). When we analysed the HLA-C antigen distribution in the articular categories of PsA, a significant association was found between the spondylitic form and HLA-Cw*0702 (OR 5.0; 95% CI 1.4 to 25.0; *p *= 0.01). HLA-B27 was found more frequently in spondylitic patients (54%) than in the groups with polyarthritis (12%) and oligoarthritis (20%) (OR 5.8; 95% CI 2.3 to 14.7; *p *< 0.0001). Uveitis cases were also more frequently found among spondylitic patients (OR 4.5; 95% CI 1.5 to 13.6; *p *= 0.006). Seventeen out of 100 patients had uveitis; of these, 10 showed the HLA-B27 antigen (59%) (OR 4.5; 95% CI 1.5 to 13.4; *p *= 0.007). However, there was no correlation between HLA-Cw*0702 and uveitis. Among PsA patients with spondylitis, those with isolated forms had the HLA-B27 antigen (92%) more frequently than those with oligoarthritis plus spondylitis (33%), and those with polyarthritis-spondylitis (37%) (*p *= 0.003). HLA-Cw*0702 was equally distributed between the three groups of spondylitis. Tables 2 and 3 show the allelic distributions between patients and controls, and between the articular subtypes of PsA.

Genetic factors have been considered to be important in studies of both susceptibility to and the expression of PsA. There are at least nine psoriasis loci, designated PSORS1 to PSORS9, and several potential loci, but the strongest association is with a locus on chromosome 6p, probably the HLA-Cw6 gene itself [2,3]. However, the pathogenic nature of these associations remains elusive. Thus, it is not clear whether HLA-Cw6 itself or a closely related gene is related to the presence of arthritis in psoriasis patients, and few studies have addressed this question so far. In a study by Gladman and colleagues, the HLA-Cw*0602 allele was increased among PsA patients, and these patients also showed an earlier age of onset of their psoriasis [8]. Another study from Poland had similar results, but also showed that patients expressing this allele had an earlier age of onset of their arthritis [9]. Nonetheless, both studies did not show whether these associations were due to the presence of psoriasis in PsA patients or, in contrast, whether it represented true associations with arthritis.

We performed HLA-C locus PCR-SSOP typing in both psoriasis and PsA patients and confirmed the known association between HLA-Cw*0602 and both entities; however, when articular subgroup comparisons were made, this allele was equally distributed between them, supporting the notion that genetic susceptibility to PsA resides in another gene. In this sense, a potential arthritogenic polymorphism of MICA (major histocompatibility complex class I chain-related gene A), called MICA-A9, has been associated with PsA risk independently of HLA-Cw6 [10].

To test the hypothesis that HLA-Cw6 might modulate some aspects of PsA in our context, we divided our PsA population into PsA Cw6-positive and Cw6-negative patients. Our findings confirmed that in Cw6-positive patients, psoriasis began at an earlier age than in Cw6-negative patients. We also found that the period between the onset of skin lesions and the appearance of arthritis was longer in Cw6-positive than in Cw6 negative-patients, but the arthritis onset age was similar in both groups, probably reflecting that other genes are responsible for arthritis onset; in that regard, the HLA-B27 antigen has been related to an earlier onset age for both psoriasis and arthritis in PsA [11].

An unexpected finding in the present study was the correlation between HLA-Cw*0702 and spondylitis. This data had not previously been reported and for that reason it deserves some consideration. The first consideration is that psoriasis and PsA show an overlapping interval of 100 kilobases between the HLA-C and OTF3 regions, which might contain the psoriasis gene, as well as arthritogenic genes including other alleles of the HLA-C locus, besides HLA-Cw6 [6]. Secondly, psoriatic spondylitis has been related to the presence of HLA-B27 in several reports worldwide (including the present study) in which this antigen might also be associated with some clinical aspects of this entity [12]. In addition, some HLAB27/Cw ancestral haplotypes may explain why some HLA-Cw antigens are over-represented in spondyloarthropathies, including PsA. According to this, the HLA-B*2705/Cw*0102, B*2705/Cw*02022 and B*2702/Cw*02022 were the main haplotypes found in normal and spondylitic patients in Spanish and Jewish populations [13]. Indeed, the spondylitic form of PsA was found in association with the HLA-B27/Cw1 haplotype in a Spanish population [14]. As we have shown here, the HLA-B27 antigen correlated well with the risk of uveitis and was more strongly associated with isolated forms of spondylitis, whereas HLA-Cw*0702 was not related to the presence of uveitis and was distributed equally between the three subgroups of spondylitis.

For the above-mentioned reasons, we hypothesize that HLA-Cw*0702 might be a new genetic risk factor for psoriatic spondylitis, in addition to the known association between it and the HLA-B27 antigen.

We found two HLA-Cw alleles under-represented in PsA patients in comparison with controls. One of them, HLA-Cw*0701, has been found as part of one of the ancestral haplotypes associated with psoriasis (HLA-Cw7-B8-DRB1*0301-DQB1*02) [15]. This finding supports our view that the role of HLA-C locus alleles in determining the risk and the clinical expression of both entities is not the same. Thus, the HLA-Cw6 antigen seems to confer a general risk for psoriasis, whereas other HLA-C alleles may act as predisposing factors for some subsets of PsA (HLA-Cw*0702 and spondylitis), and others might be 'protective' alleles (HLA-Cw*0701/Cw*0802). However, it should be borne in mind that some of these statistical associations might be lost after correction for multiple comparisons.

## Conclusion

This is the first study to show a significant association between HLA-Cw*0702 and psoriatic spondylitis, and this relationship might have an additive effect with regard to the presence of HLA-B27. The HLA-B27 antigen has a role in determining the phenotype of psoriatic spondylitis, which is not present in the HLA-Cw*0702 antigen. The potential role of HLA-Cw*0701 and HLA-Cw*0802 as 'protective' alleles in PsA should be accepted with caution, and further studies on this subject are still necessary. In contrast, genetic association studies like the present one might be hampered by the changing pattern of PsA over time, and the differences that could be found in comparison with previous studies may be consistent with the genetic distance between our population and other ethnic groups. With this in mind, larger genetic studies should be encouraged, so as to elucidate definitively the genetic basis of psoriasis and its related arthropathy.

## Abbreviations

CI = confidence interval; HLA = human leukocyte antigen; OR = odds ratio; PCR-SSOP = polymerase chain reaction-sequence-specific oligonucleotide probes; PsA = psoriatic arthritis; PSORS1 = psoriasis susceptibility 1 gene.

## Competing interests

The authors declare that they have no competing interests.

## Authors' contributions

SG reviewed the manuscript, the HLA typing and statistics, and also contributed to the writing of the last version of this manuscript. CLL reviewed the manuscript, the HLA typing and contributed to the writing of the revised version of this work. MA participated in the study design, the recruitment and evaluation of patients, and in the writing of the manuscript. CS performed the statistical design of the study and participated in the writing of the manuscript. JLR and JB participated in the writing of the manuscript and performed patient evaluations. All authors read and approved the final manuscript.

## Figures and Tables

**Table 1:** Clinical and demographic features of the study population

Variable	PsA patients (*n *= 100)
Gender ratio (M/F)	55/45
Mean age (years)	52 ± 14
Psoriasis duration (years)	24 ± 9
Arthritis duration (years)	22 ± 8
Psoriasis onset age (years)	26 ± 14
Arthritis onset age (years)	35 ± 12
Psoriasis–arthritis latency (years)	8 ± 7
Psoriasis before arthritis (%)	75
Axial pattern (%)	35
Oligoarthritis (%)	40
Polyarthritis (%)	25
DIP disease (%)	40
Mutilans form (%)	4
Onychopathy (%)	45
Erosive disease (%)	44
Enthesitis (%)	35
Uveitis (%)	17
ESR	36 ± 12
RF (%)^a^	7
ANA (%)^a^	10

ANA, antinuclear antibodies; DIP, distal interphalangeal joint; ESR, erythrocyte sedimentation rate; PsA, psoriatic arthritis; RF, rheumatoid factor. Errors are shown as SD. ^a^None of these patients had rheumatoid arthritis or systemic lupus erythematosus.

**Table 2:** Allelic distribution of HLA-C in patients and controls

HLA-C allele	Psoriasis (*n *= 45)	PsA (*n *= 100)	Controls (*n *= 177)
Cw*0102	5 (11)	10 (10)	13 (7)
Cw*0202	4 (9)	8 (8)	9 (5)
Cw*0303	3 (7)	11 (11)	19 (11)
Cw*0304	2 (4)	4 (4)	15 (8)
Cw*0401	7 (15)	23 (23)	50 (28)
Cw*0501	4 (9)	9 (9)	30 (17)
Cw*0602	26 (58)^a^	58 (58)^b^	32 (18)
Cw*0701	9 (20)	19 (19)^c^	54 (31)
Cw*0702	7 (15)	17 (17)	45 (25)
Cw*0802	2 (4)	3 (3)^d^	17 (10)
Cw*1203	3 (7)	3 (3)	6 (3)
Cw*1402	1 (2)	4 (4)	9 (5)
Cw*1502	4 (9)	5 (5)	13 (7)
Cw*1601	2 (4)	4 (4)	15 (8)
Cw*1602	-	-	2 (1)

HLA, human leukocyte antigen; PsA, psoriatic arthritis. Results in parentheses are percentages. ^a^Odds ratio (OR) 6.2 (95% confidence interval (CI) 3.1 to 12.5; *p *< 0.0001); ^b^OR 6.2 (95% CI 3.6 to 10.8; *p *< 0.0001); ^c^OR 0.5 (95% CI 0.3 to 0.9; *p *= 0.04); ^d^OR 0.3 (95% CI 0.08 to 1.00; *p *= 0.05).

**Table 3:** Allelic distributions of HLA-B27 and HLA-C among subsets of psoriatic arthritis

HLA	Oligoarthritis (*n *= 40)	Polyarthritis (*n *= 25)	Axial disease (*n *= 35)
Cw*0102	5 (12)	2 (8)	3 (9)
Cw*0202	3 (7)	2 (8)	3 (9)
Cw*0303	5 (12)	3 (12)	3 (9)
Cw*0304	2 (5)	-	2 (6)
Cw*0401	12 (30)	5 (20)	6 (17)
Cw*0501	3 (7)	3 (12)	3 (9)
Cw*0602	23 (57)	15 (60)	20 (57)
Cw*0701	8 (20)	5 (20)	6 (17)
Cw*0702	3 (7)	3 (12)	11 (31)^a^
Cw*0802	-	1 (4)	2 (6)
Cw*1203	2 (5)	-	1 (3)
Cw*1402	2 (5)	1 (4)	1 (3)
Cw*1502	3 (7)	1 (4)	1 (3)
Cw*1601	3 (7)	-	1 (3)
B27	8 (20)	3 (12)	19 (54)^b^

HLA, human leukocyte antigen. Results in parentheses are percentages. ^a^Odds ratio (OR) 5.0 (95% confidence interval 1.4 to 25.0; *p *= 0.01); ^b^OR 5.8 (95% confidence interval 2.3 to 14.7; *p *< 0.0001).
